# Hollow PdAg-CeO_2_ heterodimer nanocrystals as highly structured heterogeneous catalysts

**DOI:** 10.1038/s41598-019-55105-x

**Published:** 2019-12-11

**Authors:** Javier Patarroyo, Jorge A. Delgado, Florind Merkoçi, Aziz Genç, Guillaume Sauthier, Jordi Llorca, Jordi Arbiol, Neus G. Bastus, Cyril Godard, Carmen Claver, Victor Puntes

**Affiliations:** 1 Catalan Institute of Nanoscience and Nanotechnology (ICN2), CSIC and BIST, Campus UAB, Bellaterra, 08193 Spain; 2 Centre Tecnologic de la Química, C/Marceli Domingo, 43007 Tarragona, Spain; 3 Department of Metallurgy and Materials Engineering, Faculty of Engineering, Bartin University, 74100 Bartin, Turkey; 4 Institute of Energy Technologies, Department of Chemical Engineering and Barcelona Research Center in Multiscale Science and Engineering, Universitat Politècnica de Catalunya, EEBE, Eduard Maristany 10-14, 08019 Barcelona, Spain; 5 ICREA, Pg. Lluís Companys 23, 08010 Barcelona, Spain; 6 Departament de Química Física i Inorgànica, Universitat Rovira i Virgili, C/Marceli Domingo 1, 43007 Tarragona, Spain; 7 Vall d’Hebron Institut de Recerca (VHIR), 08035 Barcelona, Spain

**Keywords:** Nanoscience and technology, Nanoscale materials, Nanoparticles

## Abstract

In the present work, hollow PdAg-CeO_2_ heterodimer nanocrystals (NCs) were prepared and tested as catalysts for the selective hydrogenation of alkynes. These nanostructures combine for the first time the beneficial effect of alloying Pd with Ag in a single NC hollow domain with the formation of active sites at the interface with the CeO_2_ counterpart in an additive manner. The PdAg-CeO_2_ NCs display excellent alkene selectivity for aliphatic alkynes. For the specific case of hydrogenation of internal alkynes such as 4-octyne, very low over-hydrogenation and isomerization products were observed over a full conversion regime, even after prolonged reaction times. These catalytic properties were remarkably superior in comparison to standard catalysts. The promotion of Ag on the moderation of the reactivity of the Pd phase, in combination with the creation of interfacial sites with the CeO_2_ moiety in the same nanostructure, is pointed as the responsible of such a remarkable catalytic performance.

## Introduction

Since the adaptation of organic chemistry methodologies to inorganic nanocrystal (NC) synthesis a few decades ago, the research on tailoring physicochemical properties of inorganic matter by controlling the size and shape of NCs has blossomed^1^. Remarkably, the precise adjustment of NC’s properties relies on the fine adjustment of their features, and apparently, there is no restriction on the control of NC’s functionality for exploiting the unique properties of matter at the nanoscale.

In this regard, multicomponent NCs made of the combination of two or more compositional domains, each with optimized functionality, is representing a new generation of advanced functional NCs^2,3^. Despite its interest, in this design and development process several challenges and questions arise. The first is whether the excellent physicochemical properties of individual components can be maintained and combined in an additive and synergetic manner in multicomponent NCs. The second is whether current synthetic methodologies and principles can be adapted and applied to multicomponent NC achieving the same degree of control than in single component NCs. One example is the morphological and compositional control in magnetoplasmonic heterodimer NCs made of Au and Fe_3_O_4_ domains^4^. While both, the plasmonic resonance and the magnetic relaxation are affected by the presence of the other domain, their individual behavior remains basically unaltered and useful magneto-optical applications can be derived, as contrast agents for dual medical imaging^5^.

These issues are especially challenging in catalysis, where the ability to fine tailor NC’s morphological features has been translated into outstanding performance improvement and cost reduction of catalysts. An interesting case, extendedly used in many industrial areas, is the selective hydrogenation of alkynes to alkenes with marginal over-hydrogenation and isomerization issues. For this reaction, a wide variety of Pd-based catalysts have been developed such as the commonly used Lindlar, Pd poisoned with Pb supported on CaCO_3_, which provides control of the over hydrogenation or isomerization of the alkene product while maintaining high conversion rates^6^. Although widely used, it contains poisonous Pb and extensive research is being undertaken globally to find a suitable replacement^7–10^.

Here, we propose an alternative for selective hydrogenation of alkynes by producing hollow PdAg-CeO_2_ heterodimer NCs (HNCs). On the one side, the use of CeO_2_ NCs has shown special attention due to its well-known selectivity^11,12^. On the other side, the alloying of the Pd with another metal phase^13–15^, especially Pb and Ag^15–20^, allows controlling the reactivity of Pd avoiding over hydrogenation. This approach has led to new families of catalysts with outstanding properties. Stated examples are the highly selective hydrogenation of various alkynes by Au@CeO_2_^11^ and Pd@Ag@CeO_2_ NCs^12^. However, while reported examples are limited to solid structures, the use of hollow NCs^21^ has not been explored up to date although presenting multiple characteristics that enhance their catalytic activity^22,23^; they can expose very high fraction of surface sites, have strain-induced highly reactive surfaces, and allow for increased collision frequency by confining reactants within nanoscale inner cavity. Moreover, hollow NCs can result in the formation of “forbidden”alloys with interest properties in catalysis, as PtAg^24^ and require significantly lower amounts of expensive noble metals, representing a noble-metal economic design^25^. Finally, hollow structures are more robust to thermal processes than solid counterparts because surfaces dissipate the energy faster, resulting in a more stable crystal structure and a more robust catalyst.

In this context, we herein report the first example of hollow PdAg-CeO_2_ NCs catalysts that presents an outstanding selectivity for the semi-hydrogenation of alkynes. PdAg-CeO_2_ HNCs are composed by the combination of two domains, a solid one of CeO_2_, and a hollow alloyed one of PdAg which are assembled to form the heterodimeric structure. Different from a simple physical mixture where -even in close contact- the presence of topographic gaps limits the interfacial control of the system, these heterodimers maximize the metal-metal oxide interface which has been found beneficial in catalysis^26–29^. This high level of compositional control in the structure is obtained via GRR of Ag-CeO_2_ NCs, used as sacrificial templates, and K_2_PdCl_6_ used as Pd precursor. Moreover, PdAg-CeO_2_ NCs were tested in the liquid phase for the selective hydrogenation of alkynes exhibiting excellent performance in terms of selectivity and durability. This shows us both, that the strategies to address the morphology of multicomponent NCs can be easily derived from the single component NCs, and that the reduction and oxidation catalytic capacity of both domains do not cancel each other, but cooperate efficiently.

## Methods

### Reagents

Nitric acid (HNO_3_), polyvinylpyrrolidone (PVP, MW 55,000), potassium hexachloropalladate (IV) (K_2_PdCl_6_), were purchased from Sigma-Aldrich. Cerium(IV) oxide, nanopowder (15–30 nm APS Powder, S.A. 30–50 m^2^/g) was purchased from Alfa Aesar. Absolute ethanol employed as a solvent for the catalytic experiments was purchased from Merck (ACS. Iso. Reag). All chemicals were used as received without further purification. Distilled water passed through a Millipore system (ρ = 18.2 mΩ) was used in all experiments.

### Synthesis of Ag-CeO_2_ heterodimer NCs

In a typical procedure, 500 mL of 10 mM sodium citrate was added into a 1 L three-necked round bottom flask. The solution was heated until 100 °C under magnetic stirring. Before boiling started AgNO_3_ (25 mM) and Ce(NO_3_)_3_ (25 mM) were injected to the solution, and it was boiled for 4 hours. After 4 hours, the solution had an orange color, the heating was stopped, and UV-vis spectra were acquired.

### Synthesis of hollow PdAg-CeO2 heterodimer NCs

In a typical procedure, 0.5 mL of Ag-CeO_2_ NCs ([Ag^0^] = 1 mM) were added to a vial containing 1 mL PVP (5 mM) and 200 µL HNO_3_ (20 mM). 200 µL of K_2_PdCl_6_ (1 mM) was injected at a rate of 10 μL/min using a syringe pump. The reaction was stirred for 1 hour, and then the product was recovered by centrifugation (8000 g, 15 min).

### Synthesis of hollow PdAg NCs

The standard protocol for the preparation of hollow PdAg NCs consisted in two simple steps: i) First 200 mL of the Ag NPs solution (prepared by scaling up the seeded-growth method reported by Bastús *et al*.^30^ were precipitated by centrifugation and re-dispersed in 15 mL of PVP 55 K (275 mg/mL). The solution was left in a glass vial under soft stir for 24 hours to ensure PVP molecules capped all the surface of the silver templates. ii) After this 2.5 mL of HCl (250 mM) and 0.5 mL of 1 mM of K_2_PdCl_6_ were simultaneously added into the solution. After this very first injection, an additional volume of 9.5 mL of the precursor was added into the vial in 19 consecutive 0.5 mL injections with a time delay of 5 minutes between each one. During the whole process the solution is kept under vigorous stir, at room temperature, until the reaction is completed, which is indicated by the progressive change in color: from yellow (Ag NPs) to blue/purple.

### Characterization

The morphology, size and chemical composition of the NCs were visualized using FEI Tecnai G2 F20 S-TWIN HR(S)TEM, operated at an accelerated voltage of 200 kV. A droplet of the sample was drop cast onto a piece of an ultrathin carbon-coated 200-mesh copper grid (Ted-pella, Inc.) and left to dry in air. XRD data were collected on a PANalytical X’Pert diffractometer using a Cu Kα radiation source.

### Catalytic semi-hydrogenation of alkynes

The catalytic reactions were performed in a five position Par 477 autoclave equipped with glass-tubes and magnetic stirrers. In a typical experiment, each tube was charged with 5 mL of ethanol, 0.33 mmol of the substrate and the corresponding catalyst. Then the autoclaves were pressurized with 10 bar of hydrogen, and heated at 50 °C during a selected time. After the reaction, the autoclave was cooled down, de-pressurised, and the ethanolic solution analyzed by gas chromatography. The conversion and selectivities were determined using an Agilent 7890 A provided with an MS 5975 C detector using an HP5-MS column (30 m, 0.25 mm, 0.25 μm).

### X-ray Photoelectron Spectroscopy (XPS)

X-ray Photoelectron Spectroscopy (XPS) was performed on a SPECS system equipped with a monochromatic Al source operating at 300 W and a Phoibos 150 analyzer. The pass energy of the hemispherical analyzer was set at 20 eV and the energy step of high-resolution spectra was set at 0.05 eV. Binding energy (BE) values were referred to the C 1 s peak at 285.0 eV. Data processing was performed with the CasaXPS software. Cerium 3d spectra were analyzed using six peaks for Ce^4+^ (V, V″, V‴, U, U″ and U‴), corresponding to three pairs of spin-orbit doublets, and four peaks (two doublets) for Ce^3+^ (V_0_, V′, U_0_ and U′), based on the peak positions reported by Mullins *et al*.^31^, where U and V refer to the 3d_3/2_ and 3d_5/2_ spin-orbit components, respectively.

## Results and Discussion

### Synthesis of hollow PdAg-CeO_2_ heterodimer NC-based catalyst

**Figure 1:**
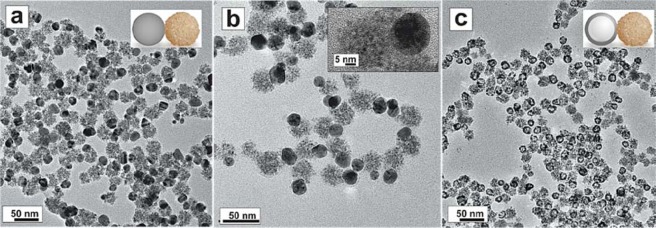
Characterization of Ag-CeO_2_ heterodimer and hollow PdAg-CeO_2_ heterodimer NCs. (**a,b**) TEM general view image of Ag-CeO_2_ heterodimer NCs, (**b**) HRTEM detail of aAg-CeO_2_ heterodimer NC -inset-, (**c**) TEM general view image of PdAg-CeO_2_ heterodimer NCs.

Colloidal solutions of highly monodisperse and well-defined hollow PdAg-CeO_2_ heterodimer NCs were produced via galvanic replacement reaction (GRR) between Ag-CeO_2_ NCs and K_2_PdCl_6_ precursor. In a first step, the Ag-CeO_2_ heterodimer NCs (Fig. 1a,b, Supplementary Fig. S1) were obtained via a modified method based on our previously reported hybrid noble metal-CeO_2_ synthesis^32,33^. The strategy relies on the rational use of sodium citrate, which plays multiple key roles, as a reducer and stabilizing agent for the initial formation of the Ag domain NCs, and as a complexing agent of Ce^3+/4+^ for its controlled oxidation and hydrolysis during the subsequent CeO_2_ deposition onto the Ag surface. Under these conditions, the homogeneous nucleation of CeO_2_ is prevented while its heterogeneous nucleation onto the Ag NCs is promoted^34^.

**Figure 2:**
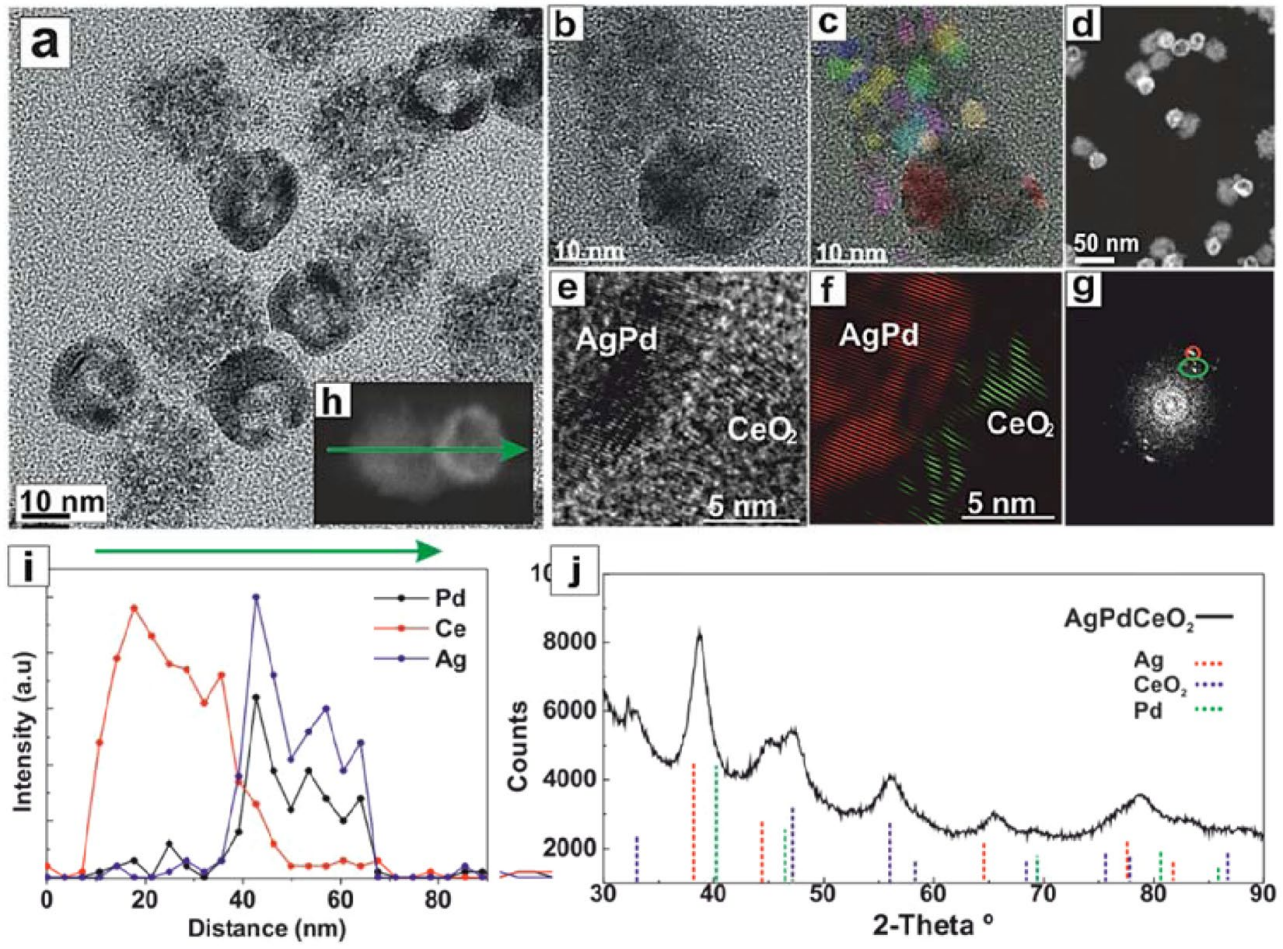
Advanced characterization of the hollow PdAg-CeO_2_ heterodimer NCs. (**a,b**) HRTEM detail of a hollow PdAg-CeO_2_ heterodimer NC, (**c**) Structural phase RGB map obtained by inverse filtering the power spectrum obtained in the HRTEM image (**d**) General HAADF-STEM image view. (**e**) HRTEM image of a heterodimer interphase (**f**) inverse FFT showing the epitaxy between PdAg and CeO_2_ NCs. (**g**) power spectrum of (**e,i**) EDS line scanning profile of the hollow PdAg-CeO_2_ heterodimer NC indicated in the HAADF STEM image detail in (**h**). (**j**). XRD patterns of hollow PdAg-CeO_2_ heterodimer NC.

Using as-obtained Ag-CeO_2_ heterodimer NCs as sacrificial templates, the precise adjustment of the synthetic parameters in the GRR provided the hollow PdAg-CeO_2_ heterodimer NCs (Figs. 1c and 2a,b). The successful conditions consisted of the slow addition of the precursor, long reaction times at room temperature, and the use of HNO_3_ as co-etcher (see Methods). The obtained structures are composed of a well-defined hollow PdAg domain (~23 nm, ~5 nm wall thickness) bound to a polycrystalline CeO_2_ phase. In Fig. 2c, we show a structural phase RGB map obtained by inverse filtering the power spectrum obtained from the HRTEM image (Fig. 2b), after mask selecting the frequency spots corresponding to different atomic plane lattices. In this way, we can visualize the random orientation of the CeO_2_ NCs. In the high angle annular dark-field scanning transmission electron microscopy (HAADF-STEM) image (Fig. 2d) the hollow PdAg domain present a brighter contrast as the higher the Z number of the atoms, higher the contrast in the HAADF-STEM and can be clearly distinguished from the CeO_2_ domain with a darker contrast (and lower average Z-density). Figure 1e shows an HRTEM image obtained from heterodimer interphase. Its corresponding power spectrum (FFT, Fig. 2g), shows diffraction spots corresponding to {111} planes of f.c.c. CeO_2_ phase (d = 0.312 nm) -marked in green- and to the {111} planes of f.c.c. PdAg phase (d = 0.236 nm) -marked in red- in the FFT. Figure 2f, present a false colored inverse FFT, revealing the pseudo-epitaxial relationship between PdAg and CeO_2_ NCs. As shown, PdAg {111} planes are almost aligned with the CeO_2_ {111} (with an out-of-plane misorientation from 10° to 15° depending on the analyzed area). In addition, and due to the different lattice parameters, there is 4 Ag {111} planes for each 3 CeO_2_ {111}. Scanning transmission electron microscopy – Energy-dispersive X-ray spectroscopy (STEM-EDS) line scan obtained on the heterodimer shown in Fig. 1h, reveals that the hollow NC’s walls are composed of a PdAg alloy (Fig. 2i). Additionally, the continuous Ce profile in the NC junction (Fig. 2h,i) constitutes a further indication of the tight interaction between PdAg and CeO_2_ domains.

The crystal structure of the hollow PdAg-CeO_2_ heterodimer NCs was further investigated by X-ray diffraction (XRD) (Fig. 2j). As shown, two series of peaks are present in the diffraction patterns of hollow PdAg-CeO_2_ NCs, the diffraction peaks at 2θ = 33.1°, 47.3° and 56.4° can be indexed to the (200), (220) and (311) fluorite (cubic) CeO_2_ phase (JCPDS 34–0394), the other peaks are attributed to the hollow bimetallic domain with a Scherrer’s crystal size of 3.2 nm for PdAg and 5.1 nm for CeO_2_. The diffraction peaks of the CeO_2_ are broad and weak, according to the small size of the CeO_2_ NCs domain. Furthermore, hollow PdAg-CeO_2_ heterodimer NCs exhibit a broader diffraction pattern with a lattice parameter of 4.03 Å. This value, lying between the two reference metals (3.859 Å for Pd-fcc and 4.079 for Ag-fcc^35^) is a further indication of the formation of an alloyed PdAg structure after the GRR process, where the Vegard’s law yields a Pd_70_Ag_30_ alloy composition.

This heterodimer nanostructure is unique, and its characteristics of relatively small particle size (in comparison to other hollow PdAg nanostructures reported to date)^36–38^ makes the obtained NCs a material with high potential in catalysis considering the extremely high surface area of the hollow PdAg domain.

### Selective hydrogenation of alkynes

The catalytic performance of the colloidal hollow PdAg-CeO_2_ heterodimer NCs was evaluated in the selective hydrogenation of alkynes in solution. The hydrogenation experiments were carried out in ethanol under 10 bar H_2_ and 50 °C. A series of terminal/internal alkynes and alkynols were tested to explore the substrate scope (Table 1).

**Table 1:** Substrate scope of selective hydrogenation of alkynes and alkynols catalyzed by the hollow PdAg-CeO_2_ heterodimer NCs catalyst and commercial references^a^.


#	Catalyst	R_1_	R_2_	Time, h	Conv, %	Sel. A, % (cis %)	Sel.B %
1	PdAg-CeO_2_	CH_3_(CH_2_)_5_	H	2	100	92 (2)	6
2	PdAg-CeO_2_	CH_3_(CH_2_)_4_	CH_3_	2	100	95 (96)	4
3	PdAg-CeO_2_	CH_3_(CH_2_)_2_	CH_3_(CH_2_)_2_	2	96	99 (94)	1
4	PdAg-CeO_2_	CH_3_(CH_2_)_2_	CH_3_(CH_2_)_2_	5	100	98 (91)	2
5	PdAg-CeO_2_	CH_3_(CH_2_)_2_	CH_3_(CH_2_)_2_	20	100	95 (85)	5
6	PdAg^c^	CH_3_(CH_2_)_2_	CH_3_(CH_2_)_2_	20	13	100 (78)	0
7	CeO_2_^d^	CH_3_(CH_2_)_2_	CH_3_(CH_2_)_2_	20	7	79 (88)	21
8	Lindlar^e^	CH_3_(CH_2_)_2_	CH_3_(CH_2_)_2_	2	100	98 (86)	2
9	Lindlar^e^	CH_3_(CH_2_)_2_	CH_3_(CH_2_)_2_	5	100	93 (74)	7
10	Lindlar^e^	CH_3_(CH_2_)_2_	CH_3_(CH_2_)_2_	20	100	79 (63)	21
11	Nanoselect^f^	CH_3_(CH_2_)_2_	CH_3_(CH_2_)_2_	2	100	0	100
12	PdAg-CeO_2_	Ph	H	2	74	88	12
13	PdAg-CeO_2_	Cy = ^b^	H	1	100	96	4
14	PdAg-CeO_2_	Ph	CH_3_CH_2_	16	13	82 (97)	18
15	PdAg-CeO_2_	Ph	CH_2_OH	16	62	84 (100)	16
16	PdAg-CeO_2_	(CH_2_)_3_OH	H	1	100	87 (1)	12

^a^Reaction conditions: Substrate (0.33 mmol), cat. (0.032 mol% Pd), 5 ml ethanol, 10 bar H_2_, 50 °C; ^b^1-ethynylcyclohexene; ^c^0.032 mol % Pd vs. 4-octyne; ^d^0.64 mol% CeO_2_ vs. 4-octyne (nanopowder, 15–30 nm); ^e^0.032 mol % Pd vs. 4-octyne; ^f^Pd Nanoselect, LF-200, 0.032 mol % Pd.

When 1-octyne was the substrate (entry 1), 92% of alkene selectivity at full conversion was observed after 2 h of reaction. Similarly, 2-octene displayed 95% of alkene selectivity (cis: trans selectivity, 96:4, entry 2) at full conversion. For the case of 4-octyne, the evolution of the reaction products during the time was studied by analyzing the crude after 2, 5 and 20 h (entries 3–5). At 2 h of reaction, the conversion was 96% and the alkene selectivity 99% (cis: trans, 94:6). At longer reaction times, the substrate was fully converted and the alkene selectivity was maintained above 95%. Even after 20 h of reaction, the overhydrogenation product did not exceed the 5%, thus evidencing the excellent resistance of the hollow PdAg-CeO_2_ heterodimer NCs against the over-hydrogenation reaction.

In order to gain insights about the source of the selectivity for this catalyst, hollow PdAg NCs with the absence of CeO_2_ NCs (diameter: 35.6 ± 3.8 nm; shell: 2.4 ± 0.4 nm; see Supplementary Fig. S2) were prepared as control and tested in the same reactions. The synthesis and the characterization of this material are described in the experimental part in the SI. The activity of the hollow PdAg NCs resulted in very low (13% conversion after 20 h, entry 6 Table 1) in comparison to hollow PdAg-CeO_2_ heterodimer NCs indicating the benefits of having the CeO_2_ as co-catalyst. Note that the presence of PVP ligand at the surface of the hollow PdAg NCs -a consequence of the different synthetic procedure to obtain PdAg NCs- may account for a mild decrease of the conversion power of the NC, but not more than a 10%^39^. In addition, a control experiment employing pure CeO_2_ NCs was also carried out (entry 7 Table 1). Under the tested conditions the activity of the pure oxide was negligible, only 7% of alkyne conversion was observed after 20 h of reaction. This observation confirms that the hydrogenation activity observed in hollow PdAg-CeO_2_ heterodimer NCs can be attributed to the combined action of the hollow PdAg NCs with the CeO_2_ component. Indeed, the electronic and geometric effect of the alloying of Pd with Ag can moderate the catalyst activity preventing the formation of sub-surface hydrides which are generally responsible for over-hydrogenation issues in palladium-based catalysts operating above atmospheric pressure^40^. Secondly, a synergic effect between CeO_2_ and the PdAg structure may impact positively in the catalyst selectivity as reported for other CeO_2_ containing hybrid nanocatalysts^11^.

For comparison purposes, two commercial references, the Lindlar, and Pd Nanoselect catalysts were also tested in the hydrogenation of 4-octyne under the same conditions employed for the heterostructured PdAg-CeO_2_ NCs (entries 8–10 vs. 3–5). The Lindlar catalyst displayed full conversion of the alkyne after 2 h of reaction (entry 8). It is noteworthy that both the alkene and the *cis* selectivity decreased from 98 to 79% and 86 to 63% when the reaction was continued for 20 h (entry 8 vs. 10). This observation suggests that under the tested conditions, Lindlar catalyst (in the absence of nitrogenated additives) does not prevent overhydrogenation or isomerization processes as well as the hollow PdAg-CeO_2_ heterodimer NCs even after 20 h of reaction (entries 5 and 10). For the present case, it is evident the higher resistance of PdAg-CeO_2_ against isomerization or over-hydrogenation reactions at extended reaction times. Considering that the Pd loading was the same for both tests (0.032 mol% Pd), diverse reactivities are reasonably justified by the differences of not only composition but also nanostructure. Similarly, the commercial Pd Nanoselect LF-200 catalyst was tested in the hydrogenation of 4-octyne (entry 11). Under the tested conditions, full overhydrogenation of the alkyne occurred after 2 h of reaction. This observation evidences the positive effect of alloying the Pd phase with silver for the moderation of the hydrogenation activity at the same time that the amount of the noble metal is optimized in the porous hollow structure. Indeed, the effect of silver in PdAg bimetallic systems for the selective hydrogenation of alkynes is widely documented in the literature^18,41,42^. Regarding other terminal alkynes and alkynols (entries 1, 12, 13, 16), relatively short reaction times (<2 h) were required in general to complete the semi-hydrogenation. From the tested terminal alkynes, phenylacetylene displayed the lowest conversion (74% after 2 h, entry 12). Curiously, internal alkynes substituted with phenyl rings (entries 14 and 15), displayed remarkably low conversions (13–60%) even after prolonged reaction times (16 h). Additionally, in spite of their low conversions, these compounds suffered from marked overhydrogenation issues. Considering that internal alkynes such as 4-octyne did not evidence such a slow conversion or premature overhydrogenation, we believe that a sum of electronic/steric effects of the phenyl ring on the triple bond (e.g. charge delocalization) in combination with their particular adsorption properties at the surface of the hollow PdAg-CeO_2_ heterodimer NCs, is the responsible for the observed reactivity constraints.

### X-ray photoelectron spectroscopy

**Figure 3:**
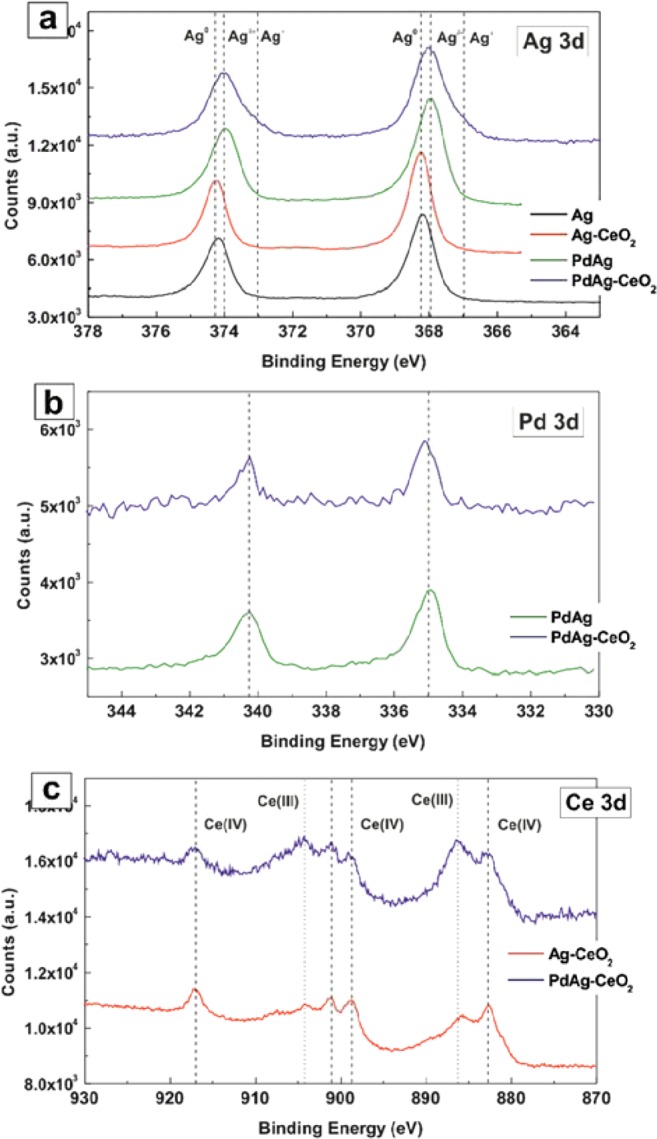
XPS spectra of the as-synthesized Ag, Ag-CeO_2_, hollow PdAg and hollow PdAg-CeO_2_ NCs: (**a**) Ag 3d, (**b**) Pd 3d, (**c**) Ce 3d.

To evaluate the electronic structure of the hollow PdAg-CeO_2_ heterodimer NCs and related controls, we performed X-ray photoelectron spectroscopy (XPS) measurements. Ag 3d, Pd 3d and Ce 3d spectra of all samples are shown in Fig. 3a–c, respectively. In the case of Ag solid NCs, only a doublet is observed corresponding to the well-known spin-orbit splitting. The binding energy (BE) at 368.2 and 374.2 eV, assigned to 3d5/2 and 3d3/2, respectively, corresponds well to metallic Ag. Essentially, the same spectrum is obtained in the case of Ag-CeO_2_ heterodimer NCs, suggesting that there is no significant electron transfer between the Ag and CeO_2_ domains. On the contrary, a clear chemical shift of the Ag 3d peaks, now located at 368.0 and 374.0 eV can be seen in the hollow PdAg alloy sample. This chemical shift toward lower binding energy values indicates partial oxidation of Ag, which results from electron transfer from Ag to Pd. This can be explained in terms of the differences in electronegativity between both metals; Pd is more electronegative than Ag.

Accordingly, the Pd 3d5/2 and 3d3/2 signals exhibit BE values slightly lower in energy than metallic Pd (Fig. 3b), which is in accordance with the electron transfer from Ag to Pd in the hollow PdAg alloy NCs. Remarkably, the bands in the hollow PdAg alloy are broader than those of Ag which can be explained in terms of the thickness and unique morphology of the PdAg walls in the hollow structure. Interestingly, the hollow PdAg-CeO_2_ sample shows additional bands in the Ag 3d spectrum with another doublet at 367.0 and 373.1 eV. These signals, displaced towards lower binding energy values, indicate electron transfer from Ag in PdAg to CeO_2_ and can be ascribed to a particularly strong interaction between PdAg and CeO_2_. Taking into account that these bands are not observed in the Ag-CeO_2_ sample, it can be concluded that a particular electronic rearrangement between the PdAg alloy and the CeO_2_ domain in the PdAg-CeO_2_ heterodimer NCs takes place, which accounts for the particular catalytic performance observed.

This electron donation from PdAg to CeO_2_ is also evidenced when the Ce 3d spectra of PdAg-CeO_2_ and Ag-CeO_2_ samples are compared (Fig. 3c). The amount of Ce(III) species in the hollow PdAg-CeO_2_ sample is much higher than in Ag-CeO_2_. On the contrary, in the hollow PdAg-CeO_2_ sample, the Pd 3d5/2 and 3d3/2 signals appear at a slightly higher BE values than PdAg, 335.1 and 340.4 eV, which is consistent with an electron transfer from PdAg to CeO_2_. These electronic effects on PdAg NCs in terms of charge transfer from Ag to Pd have been well documented in the literature, and such an effect has been directly correlated with the enhancement of the alkene selectivity in the semi-hydrogenation of alkynes, due to modification of adsorption energies of reagents and products from the particles surface^43–45^. For instance, Kang el at reported the existence of charge transfer between Ag and Pd^44^. Likewise, Huang *et al*.^45^ observed an improvement in the selectivity on Ag-promoted Pd catalysts and ascribed such an effect to the increase in the Pd d-band electron density by the addition of Ag.

## Conclusions

In summary, hollow PdAg-CeO_2_ heterodimer NCs were tested as catalysts for the selective hydrogenation of alkynes. The NC is prepared after the GRR of the Ag counterpart of an Ag-CeO_2_ heterodimer and a Pd salt. These NCs were used for the selective hydrogenation of alkynes. Hydrogenation is one the most extensively applied catalytic process in the chemical industry, with a continuously increasing application scope, such as multistep synthesis of fine chemicals and pharmaceuticals. For the specific case of internal alkynes such as 4-octyne, excellent alkene selectivity was obtained with marginal over-hydrogenation and isomerization issues over a full conversion regime even after prolonged reaction times. These properties were remarkably superior in comparison to commercial Pd based catalysts and were attributed to a combination of the promotional effect of Ag on the moderation of the reactivity of the Pd phase and a possible synergic effect between the CeO_2_ and the hollow nanostructure. The hollow nature of the PdAg domain, optimizes also the exposed surface area of the active phase, thus saving the requirement of expensive noble metals. The reported structure constitutes a step forward towards the next generation of catalysts conceived from nanoengineering as it is clear that NC design can be made additive and that current synthetic strategies can be applied to obtain multicomponent NCs with desired architectures.

## Supplementary Information

Supplementary Information
