# Supplementary material for: Hollow PdAg-CeO2 heterodimer nanocrystals as highly structured heterogeneous catalysts
Source: Sci Rep. 2019 Dec 11;9:18776. doi: 10.1038/s41598-019-55105-x (PMC6906419; doi:10.1038/s41598-019-55105-x)
Supplement: Supplementary file 1 — Supplementary Information [file 41598_2019_55105_MOESM1_ESM.pdf]

Supplementary Information

Hollow PdAg-CeO_2 heterodimer nanocrystals as highly structured heterogeneous catalysts.

Javier Patarroyo, Jorge A. Delgado, Florind Merkoçi, Aziz Genç, Guillaume Sauthier, Jordi Llorca, Jordi Arbiol, Neus G. Bastus, Cyril Godard, Carmen Claver and Victor Puntes.

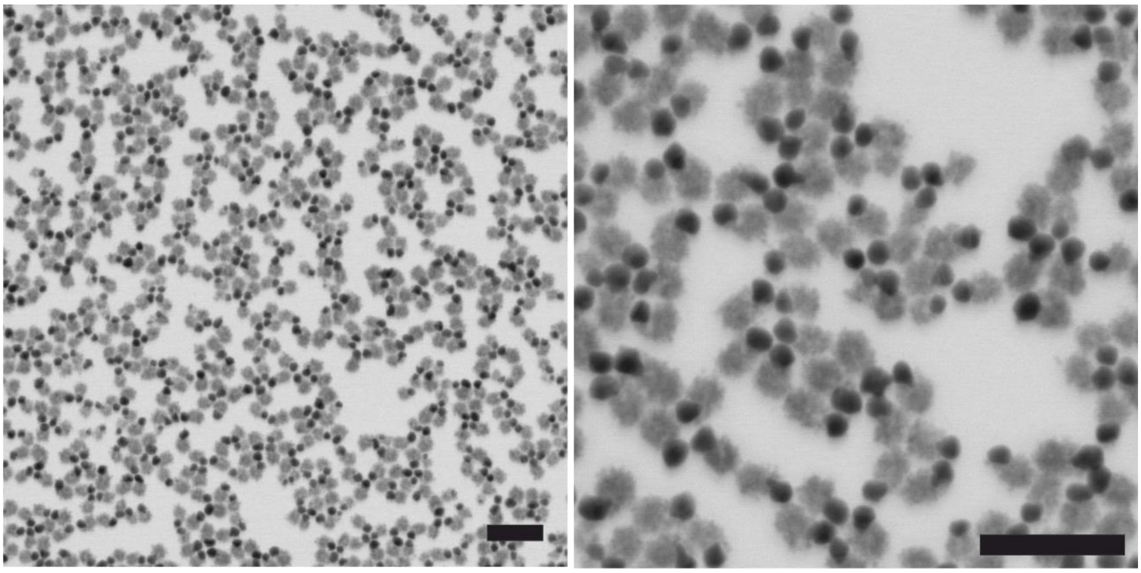

Figure S1. TEM images of Ag-CeO_2 heterodimer NCs. Scale bars represents 100 nm for all images.

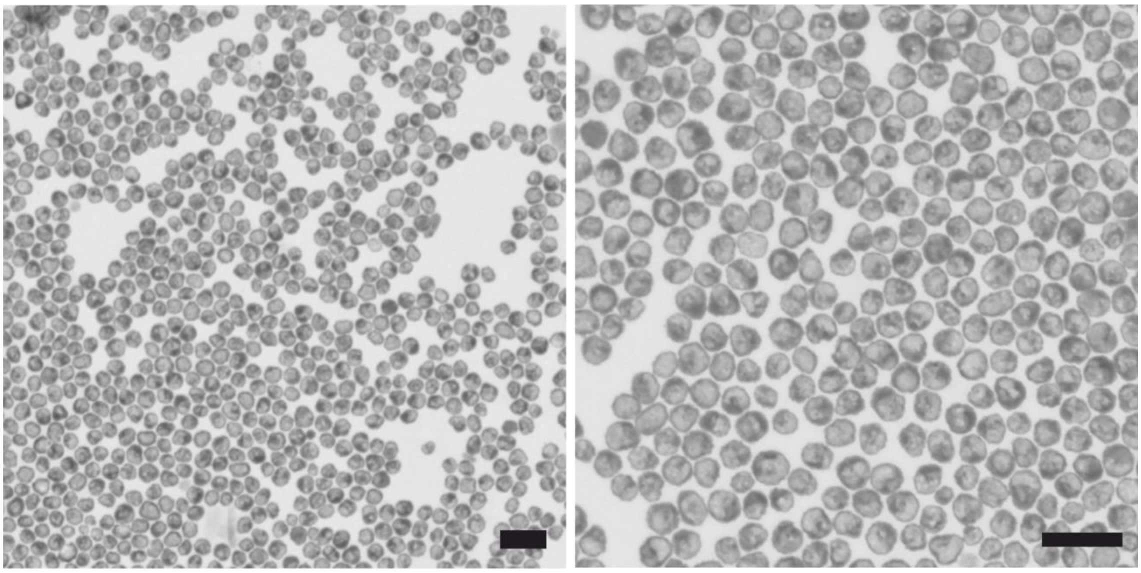

Figure S2. TEM images of hollow PdAg NCs. Scale bars represents 100 nm for all images.
